# Altered offspring neurodevelopment in an arginine vasopressin preeclampsia model

**DOI:** 10.1038/s41398-021-01205-0

**Published:** 2021-01-28

**Authors:** Serena Banu Gumusoglu, Akanksha Sri Satya Chilukuri, Benjamin Wen Qing Hing, Sabrina Marie Scroggins, Sreelekha Kundu, Jeremy Anton Sandgren, Mark Kharim Santillan, Donna Ann Santillan, Justin Lewis Grobe, Hanna Elizabeth Stevens

**Affiliations:** 1 Interdisciplinary Neuroscience Graduate Program, University of Iowa, Iowa City, IA USA; 2 Department of Psychiatry, Carver College of Medicine, University of Iowa, Iowa City, IA USA; 3 Iowa Neuroscience Institute, University of Iowa, Iowa City, IA USA; 4 Molecular Physiology and Biophysics, Carver College of Medicine, University of Iowa, Iowa City, IA USA; 5 Department of Obstetrics and Gynecology, Carver College of Medicine, University of Iowa, Iowa City, IA USA; 6 Department of Pharmacology, Carver College of Medicine, University of Iowa, Iowa City, IA USA; 7 Department of Physiology, Medical College of Wisconsin, Milwaukee, WI USA

**Keywords:** Molecular neuroscience, Physiology

## Abstract

Preeclampsia is a severe gestational hypertensive condition linked to child neuropsychiatric disorders, although underlying mechanisms are unclear. We used a recently developed, clinically relevant animal model of preeclampsia to assess offspring. C57BL/6J mouse dams were chronically infused with arginine vasopressin (AVP) or saline (24 ng/h) throughout pregnancy. Adult offspring were behaviorally tested (Y-maze, open field, rotarod, social approach, and elevated plus maze). Offspring brain was assessed histologically and by RNA sequencing. Preeclampsia-exposed adult males exhibited increased anxiety-like behavior and social approach while adult females exhibited impaired procedural learning. Adult AVP-exposed males had reduced total neocortical volume. Adult AVP-exposed females had increased caudate–putamen volume, increased caudate–putamen cell number, and decreased excitatory synapse density in hippocampal dentate gyrus (DG), CA1, and CA3. At postnatal day 7 (P7), AVP-exposed male and female offspring both had smaller neocortex. At P7, AVP-exposed males also had smaller caudate–putamen volume, while females had increased caudate–putamen volume relative to neocortical size. Similar to P7, E18 AVP-exposed offspring had smaller dorsal forebrain, mainly in reduced intermediate, subventricular, and ventricular zone volume, particularly in males. Decreased volume was not accounted for by cell size or cerebrovascular vessel diameter changes. E18 cortical RNAseq revealed 49 differentially-expressed genes in male AVP-exposed offspring, over-representing cytoplasmic translation processes. In females, 31 genes were differentially-expressed, over-representing collagen-related and epithelial regulation pathways. Gene expression changes in E18 AVP-exposed placenta indicated potential underlying mechanisms. Deficits in behavior and forebrain development in this AVP-based preeclampsia model were distinctly different in males and females, implicating different neurobiological bases.

## Introduction

Preeclampsia is a gestational hypertensive condition with no corrective treatment aside from delivery. While antihypertensive drugs do prevent stroke, they do not prevent other preeclampsia sequelae^1,2^. Preeclampsia exposure is linked to neuropsychiatric outcomes in exposed children^3^, including increased rates of autism spectrum disorder (ASD), attention deficit hyperactivity disorder, and cognitive deficits^4–10^. Furthermore, preeclampsia-exposed children exhibit altered brain tractography, resting-state functional connectivity, and cerebrovasculature^11–13^. Despite an epidemiological connection between preeclampsia and altered offspring neurodevelopment, the underlying mechanism by which offspring neurobiology and behavior is altered is poorly understood.

Multiple animal models have been developed to uncover preeclampsia mechanisms. One unique mouse model of preeclampsia involves continuous maternal administration of arginine vasopressin (AVP) throughout pregnancy^14,15^. AVP secretion is elevated in human preeclampsia, and AVP is sufficient to recapitulate clinically-significant features of preeclampsia in mice (e.g., proteinuria, glomerular endotheliosis, pregnancy-dependent/progressive gestational hypertension, inflammation)^14–16^. Critical advantages of this model are that it causes pregnancy-specific hypertension and requires no potentially confounding surgical manipulation during gestation. Despite translatable effects on the fetus and placenta (e.g., intrauterine growth restriction, adverse placental outcomes), the impact of increased maternal AVP on offspring neurodevelopment remains poorly understood and is the focus of this study.

To determine any impacts of this published model on offspring neurodevelopment, we assessed offspring behavior in adulthood and neurobiology at multiple timepoints. We hypothesized that chronic prenatal maternal AVP in mice would recapitulate preeclampsia-like outcomes in children, including altered behavior and neurodevelopment. These assessments may lead to critical mechanistic insights into the targeted treatment and/or prevention of preeclampsia neurodevelopmental outcomes in clinical populations.

## Materials and methods

### Mice and infusions

C57BL/6J female mice (Jackson, Bar Harbor, ME) were randomized to one of two conditions (saline or AVP) and subcutaneously implanted with osmotic minipumps (#1004, Alzet, Cupertino, CA), loaded with either AVP (subcutaneously released at 24 ng/h; PeproTech, London, UK) or 0.9% sterile NaCl (saline; subcutaneously released at 0.11 µL/h), three days prior to breeding, as previously described^15^. Prior to implantation, pumps were primed with submersion in 23 °C saline for 12 h. Detection of a vaginal plug was designated as embryonic day (E)0. At E0, dams were individually housed. A final 26 dams were used for all end points across ages. To reduce variability in handling, a single unblinded experimenter handled animals throughout.

Proteinuria in the dam was assessed using the Pierce BCA assay kit (Thermofisher), as previously described^15^. Offspring were separated by sex and group-housed after weaning. All experimental procedures involving animals were approved by the University of Iowa Institutional Animal Care and Use Committee.

### mRNA sequencing (mRNA-seq)

Total RNA from four brains per sex per condition (16 total samples from 8 litters) was extracted using the RNeasy Mini Kit (Qiagen, Hilden, Germany). Four biological replicates sufficiently powers detection of differentially-expressed genes^17,18^. RNA integrity (RNA 6000 Nano kit, Agilent, Santa Clara, CA; 2100 Bioanalyzer Tapestation, Agilent) (RIN ≥ 8) was also ensured. mRNA-seq library preparation (TruSeq® Stranded mRNA Library Prep, Illumina, Madison, WI) was tested using the DNA 1000 kit (Agilent) and 2100 Bioanalyzer Tapestation, and 150 bp paired-end reactions across two lanes were used to sequence libraries (HiSeq 4000, Illumina).

### Quantitative polymerase chain reaction

Placenta, including all placental layers, (one per sex from each of three litters per condition), and E18 dorsal forebrain were processed for total mRNA (RNeasy Mini Kit, Qiagen). Total RNA concentration was determined (Nanodrop Spectrophotometer, Thermofisher) and 1 μg was reverse transcribed to complementary DNA (AMV First Strand cDNA Synthesis kit, BioLabs, Ipswich, MA). Samples were run in triplicate (ViiA 7 Real-Time PCR System, Thermofisher), and gene expression calculated from average Ct values normalized to 18 S rRNA for placenta and GAPDH for brain [formula: 2^(−ΔCt)^]. SYBR Green primers were used (Supplementary Table 1).

### Immunohistochemistry

Brains (*n* = 5 adult and P7 brains per sex, condition; *n* = 6 E18 brains per sex, condition except DAPI cell size measures, where *n* = 5 per sex, condition) were dissected, fixed (4% PFA), and cryoprotected (20% sucrose) prior to cryo-sectioning (adult: 50 µm; P7: 30 µm; E18: 25 µm). Sections were immunostained as previously^19^ with primary antibodies for S100β (1:500, cat # SAB2108392, lot # QC3278, Sigma-Aldrich), NeuN (1:300, cat # 24307 S, lot # 4, Cell Signaling Technology, Danvers, MA), Tbr1 (1:500, cat # AB31940, lot # GR3197859-1, Abcam, Cambridge, UK), PSD95 (1:400, cat # 36233, Cell Signaling Technology, Danvers, MA), and vGlut1 (1:1000, cat # AB5905, lot # 2840464, EMD Millipore, Burlington, MA). Sections were then incubated with appropriate secondary antibodies (1:500; Molecular Probes, Eugene, OR). Cerebrovasculature was visualized using Isolectin B4 (IB4) conjugated to Alexa Fluor 488 (Thermofisher). All sections were mounted with DAPI mounting medium (Vector Laboratories, Burlingame, CA).

### Stereology

Cell numbers were assessed via unbiased stereology, as described previously^20^. StereoInvestigator software (Microbrighfield, Colchester, VT) was coupled to a Zeiss AxioImager 2 (Carl Zeiss, Oberkochen, Germany) with a digital camera and motorized stage, and was used for all assessments of cell numbers, cell densities, and regional volumes. Cells were assessed in predefined counting frames within randomly generated grids using Stereoinvestigator’s “optical fractionator” workflow. For volumetric assessments, embryonic dorsal forebrain included the primordial hippocampus, cortical plate, and ventricular, subventricular, and intermediate zones, while adult cortex included all six cortical layers. Embryonic cortical plate volume was determined using TBR1 immunolabeling. To determine the volume of the E18 dorsal forebrain intermediate, subventricular, and ventricular zones together (IZ + SVZ + VZ), the difference between total dorsal forebrain and TBR1 + cortical plate volume was calculated for each individual. P7 and adult brain regions were assessed using the publicly available Allen Brain Atlas (www.brain-map.org). Colocalized Vglut1 and PSD95 puncta were assessed using ImageJ’s “Colocalization” plugin^21^. Cell and vessel diameters were measured using ImageJ’s tracing function. Averages from a minimum of three sections per brain were used for cell and vessel diameters (~20 cells or vessels per section).

### Behavior

Adult offspring (~8 weeks old) were assessed on one task per day during their light cycle after 30 min of daily habituation to the testing room. Tissues were collected at least 2 weeks after behavioral testing.

#### Three-chamber social approach

Male mice (*n* = 10 saline less one outlier in time per interaction assessment, 14 AVP) were habituated (5 min) to the center chamber of a three-chambered social approach apparatus, which was flanked by two side chambers, each containing an inverted metal mesh cup (cup bars ~1-cm apart). Mice were then allowed access to all three chambers for a 10-min testing period. During testing, a male, age-matched conspecific “stranger” mouse was confined to one mesh cup (chamber side counterbalanced between tests). Only male mice were tested due to logistical constraints. The other cup remained empty. Stranger mice had no prior interactions with test mice and were used twice daily at maximum. A blinded experimenter coded all forward-facing interactions with either cup in which the test mouse’s nose passed between cup bars. Summed total interactions with either cup assessed exploration and interaction ratio (social cup: social and empty cup) assessed social preference. Time per cup interaction was calculated using Anymaze software (Wood Dale, IL) coupled to an overhead USB camera.

#### Elevated plus maze

Mouse time spent on the open arm of a standard elevated plus maze apparatus was assessed (5 min; *n* = 11 saline less one outlier, 13 AVP males; 12 saline less one outlier, 14 AVP females) using Anymaze software.

#### Open field

Mouse locomotor activity was assessed using a plexiglass rectangular open field (~1800 cm^2^). Total distance traveled was assessed over 30 min (Anymaze; *n* = 11 saline, 14 AVP males; 12 saline, 13 AVP females less one outlier for total distance traveled).

#### Rotarod

Mice (*n* = 11 saline, 14 AVP males; 13 saline less one outlier, 12 AVP females) were tested over four trials (~45 min between each) on a single day using the rotarod apparatus (Stoelting). Mice navigated a horizontal, rotating, textured rod suspended above a platform which automatically detected and recorded animal fall latencies. Rod rotation mechanically accelerated from 4 rotations per minute (rpm) to 80 rpm over 4 min. Latency to fall from the rod as it accelerated was assessed during the first (pretraining) and last (post-training) two trials.

#### Y-maze

Animals (*n* = 12 saline, 14 less one outlier AVP males; 12 saline less one outlier, 14 AVP females) were placed into the center of an opaque plexiglass, three-armed (120° apart and joined at the center) apparatus. Arms were enclosed with side walls and mouse movement in 5 min recorded overhead. A spontaneous alternation ratio was calculated as complete alternations through three arms in sets of three entries (e.g., arm A to B to C but not arm A to B to A) over total three-entry sets.

### Statistical analyses

Two-way ANOVAs and Bonferroni’s multiple comparisons tests were used to analyze animal weights (examining effects of exposure and sex) and dimensions of social behavior (examining effects in different zones); corrected *p* values are reported for multiple comparison tests, as appropriate. Stages of the rotarod were assessed independently to reflect the effects of treatment on each individual phase (pre and post-training). Nonparametric litter size and resorption data were compared by the Mann–Whitney test. For other comparisons, males and females were assessed independently by two-sided, two-sample *t*-tests and the assumption of homogeneity of variances was met. Statistical analyses were independent by sex given the a priori hypothesis that neurodevelopmental and related effects of AVP exposure would be sex-dependent, as most neurodevelopmental disorders exhibit significant sex differences. This is also supported by sex-specific offspring assessments in the neuropsychiatric and maternal exposure literature^22,23^. Pearson’s coefficients with two-tailed significance corrected for multiple comparisons were calculated between behavioral and adult neurobiological measures. Statistical outlier limits were calculated (greater than two standard deviations from the mean) and outliers excluded. *P* values <0.05 were significant and <0.1 were trends. Plotted averages depict means ± S.E.M. Sample sizes were selected based on our prior work in similar, gestational exposure models^19,20,24^.

For mRNA-Seq analyses, Illumina adapters were removed using Trim Galore (version 0.4.1) and verified by FastQC (version 0.5.2). Data were quasi-mapped to GENCODE version M16 GRCm38 (mm10) assembly and quantified using parameters –bias –bootstrap-samples = 200 –rf-stranded in Kallisto (version 0.43.1)^25–27^. The average mapping efficiency (~89%; average 28,335,347 mapped reads per sample) determined here was consistent with the literature^25,27^. Read data were pooled across lanes (average reads/sample: 31,707,708). Differential gene expression was determined using high sensitivity, qPCR-validated methods (Sleuth version 0.29.0 pipeline in R statistical computing)^28,29^. Estimates of transcript abundance (Kallisto) were normalized to gene length for gene level analysis. A principle component analysis approach was used to assess variance between samples and resultant data analyzed using a linear model (scaled reads per base as the response variable, treatment group as the explanatory variable). Males and females were analyzed separately (*n* = 4 per group per sex except *n* = 3 for female AVP, excluding one outlier for high variance). Statistical differences between treatment groups (false discovery rate <0.05) were calculated via the Wald test, with beta values (Sleuth) as effect sizes. For functional annotation, significantly changed genes (*p* < 0.05) were used as input for PANTHER (version 15.0) GO-Biological Process binomial overrepresentation testing^30^. RNA sequencing data are publicly available at GEO (GSE160676).

For a secondary analysis of previously-published E12.5 placental mRNA-seq results^15^ for ASD candidate gene overrepresentation, differentially-expressed transcripts were compared to Simons Foundation for Autism Research Initiative (SFARI) Human Genes (835 score 1–3 genes and 78 syndromic genes, downloaded March 24, 2020) using enrichment testing via the one-tailed Fisher’s exact test in R Studio.

## Results

### Gestational and offspring growth outcomes

As previously reported^15^, AVP-infused dams had higher urine protein at E18 [mean ± SEM; saline: 1.10 ± 0.23 ng/μL, AVP: 3.00 ± 0.50 ng/μL; *t*(9)=3.66, *p* = 0.005)], indicating the renal dysfunction that is cardinal to preeclampsia^31^. Litter size (median, range; saline: 7, 5–8; AVP: 7, 5–9) and resorptions at E18 (saline: 0, 0–4; AVP: 1.5, 0–3) were unchanged by AVP infusion. Additionally, no notable dam morbidity, mortality, or other gestational complications occurred.

Offspring body size at E14 was unaffected by maternal AVP infusion (Supplementary Fig. 1A), but AVP-exposed offspring weighed less at E18 [main effect of AVP by two-way ANOVA *p* < 0.001, *F*_1,58_ = 15.10); AVP-exposed E18 males weighed significantly less and females trended smaller [posthoc *t*-test males: *t*(36) = 3.375, *p* = 0.0018; females: *t*(22) = 2.275, *p* = 0.033, corrected alpha = 0.025] (Supplementary Fig. 1B). By P21, female AVP-exposed offspring weighed 9.7% more [interaction by two-way ANOVA *p* = 0.029, *F*_1,50_ = 5.069; posthoc *t*-test females: *t*(26) = 2.747, *p* = 0.011; males: *t*(24) = 0.753, *p* = 0.46, corrected alpha = 0.025] (Supplementary Fig. 1C), a phenotype that persisted into adulthood when females weighed 6.2% more and males remained unchanged [main effect of AVP by two-way ANOVA *p* = 0.0097, *F*_1,46_ = 7.286; posthoc *t*-test females: *t*(23) = 3.926, *p* = 0.0007; males: *t*(23) = 1.144, *p* = 0.26, corrected alpha = 0.025] (Supplementary Fig. 1D).

### Adult offspring behavior

**Fig. 1:**
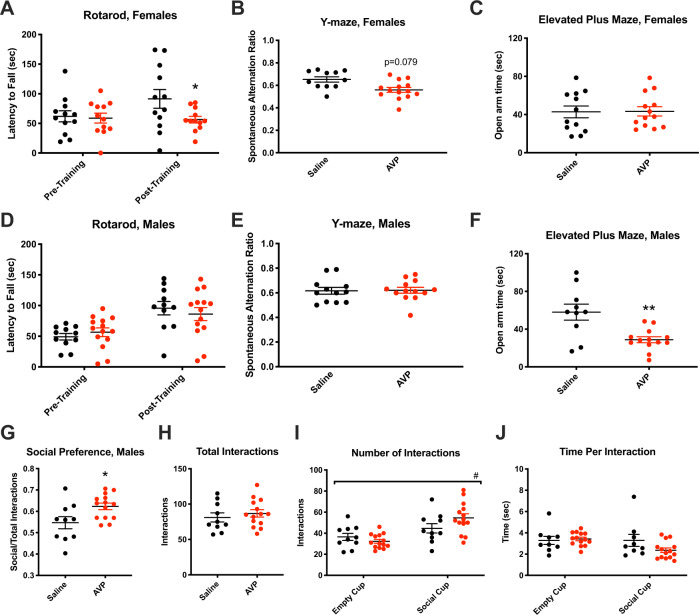
Adult offspring behavior was sex-specifically changed by prenatal maternal AVP. **a** AVP-exposed adult females exhibited decreased post-training performance on the rotarod procedural learning task (*p* = 0.048), while (**d**) male rotarod learning was unchanged by AVP status. **b** Trend-significant working memory deficits, as measured by spontaneous alternations on the Y-maze, also occurred in AVP-exposed female (*p* = 0.079), but not male (**e**), offspring. **c** While AVP-exposed female did not exhibit anxiety-like behavior changes, **f** AVP-exposed male offspring spent significantly decreased time on the open arm of the elevated plus maze (*p* = 0.0019). **g** Social preference was significantly (*p* = 0.018) increased in AVP-exposed male offspring, while (**h**) total interactions were unchanged. **i** Offspring from both conditions had significantly more interactions with the social cup than with the empty cup [main effect of cup type by two-way ANOVA *p* < 0.0001, *F*_1,44_ = 19.31], while (**j**) time spent at each cup did not differ by condition. ^#^*p* < 0.0001 by two-way ANOVA, *p < 0.05, **p < 0.005 per two-sample *t*-test, error bars represent SEM. 4 litters per condition, 1–6 offspring per sex per litter.

AVP-exposed female, but not male, offspring exhibited decreased rotarod procedural learning relative to controls [females: *t*(22) = 2.095, *p* = 0.048] (Fig. 1A, D). Critically, there were no differences among males or females in pre-training performance (Fig. 1A, D). Procedural learning deficits were paired with trend-wise working memory deficits in AVP-exposed female, but not male, offspring, evidenced by trend-decreased Y-maze spontaneous alternation [females: *t*(10) = 1.953, *p* = 0.079] (Fig. 1B, E).

AVP-exposed male, but not female, offspring exhibited increased anxiety-like behavior, demonstrated by decreased time in the open arm of the elevated plus maze [*t*(21) = 3.554, *p* = 0.0019] (Fig. 1C, F). Open field behavior (e.g., time spent in the center of the field, total distance traveled) did not differ between groups (Supplementary Fig. 2).

To assess social behavior, the three-chamber social preference task was employed in male offspring. AVP-exposed male offspring exhibited increased sociability relative to controls [*t*(22) = 2.547, *p* = 0.0184] (Fig. 1G). The total number of interactions with both cups did not differ between groups (Fig. 1H), and both saline- and AVP-exposed offspring had more total interactions with the social than empty cup (main effect of cup type by two-way ANOVA *p* < 0.0001, *F*_1,44_ = 19.31) (Fig. 1I). Time spent per interaction with each cup did not differ between groups (Fig. 1J).

### Adult offspring neurobiology

Given robust, sex-specific changes in adult offspring behavior after gestational AVP, we next examined whether adult neurobiology was impacted in males and females. Adult cortical volume, total cell density, neuronal density, and macroglia (astrocytes and oligodendrocytes) densities were not changed by AVP in male or female offspring (Supplementary Fig. 3A–D). Likewise, prefrontal cortex, corpus callosum, and hippocampal (DG and CA) volumes were unchanged (Supplementary Fig. 3E–H).

**Fig. 2:**
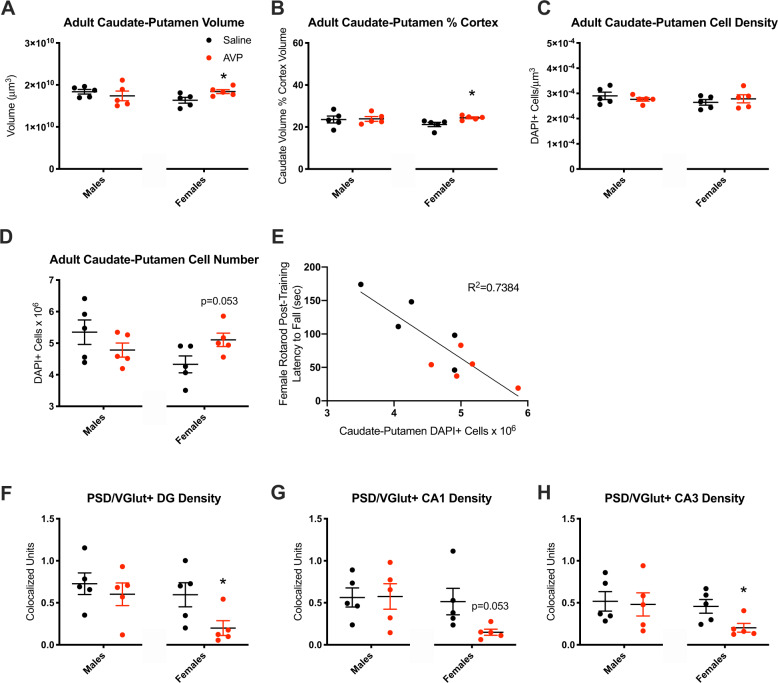
Adult offspring neurobiology was altered by maternal AVP. **a** AVP-exposed females had increased caudate–putamen volume (*p* = 0.037), (**b**) as well as increased caudate–putamen volume relative to cortex volume (*p* = 0.021). **c** Caudate–putamen cell density was unchanged. **d** The number of cells in the caudate–putamen was trend-increased in AVP-exposed females (*p* = 0.053). **e** Caudate–putamen cell numbers were inversely correlated with post-training rotarod performance in adult female offspring (*R* = −0.6809; *p* = 0.0014 by exploratory analyses; *q* = 0.0056 after Benjamini–Hochberg FDR correction). **f** Excitatory synapse density (PSD95/VGlut1 colocalization) was decreased in the DG in AVP-exposed females (*p* = 0.046), and (**g**) trend-decreased in the CA1 (*p* = 0.053) and decreased in the (**h**) CA3 (*p* = 0.030) regions. *p < 0.05 per two-sample *t*-test, error bars represent SEM. 2–3 litters per condition, 1–3 brains per sex per litter.

Despite no changes elsewhere, caudate–putamen volume was increased only in AVP-exposed females [*t*(8) = 2.498, *p* = 0.037], similar to female-specific procedural learning deficits. Caudate–putamen volume relative to cortical volume was also increased only in female AVP-exposed offspring [*t*(8) = 2.873, *p* = 0.021] (Fig. 2A, B). Caudate–putamen cell density was unchanged, such that total cell number was trend-increased in AVP-exposed females relative to controls [*t*(8) = 2.274, *p* = 0.053] (Fig. 2C, D). Female post-training rotarod performance was negatively correlated with caudate–putamen cell number (Fig. 2E) [*F*(8) = 22.58, *R* = −0.6809; *p* = 0.0014 by exploratory analyses; *q* = 0.0056 after Benjamini–Hochberg FDR correction].

Given abnormalities in memory-related behavior in female AVP-exposed offspring, we also assessed excitatory synapse density in memory-related brain areas—the hippocampal DG, CA1, and CA3 and prefrontal cortex. This revealed a decreased density in the DG only in AVP-exposed females [*t*(8) = 2.356, *p* = 0.046] (Fig. 2F). PSD95/VGlut1 colocalized puncta density in AVP-exposed females was likewise trend-decreased in CA1 [*t*(8) = 2.263, *p* = 0.053] and decreased in CA3 [*t*(8) = 2.630, *p* = 0.030] (Fig. 2G, H). Unlike the hippocampus, PSD95/VGlut1 colocalized puncta density in the prefrontal cortex was unchanged (male saline: 0.488 ± 0.067, AVP: 0.368 ± 0.041; female saline: 0.470 ± 0.056, AVP: 0.451 ± 0.041).

### Juvenile offspring neurobiology

**Fig. 3:**
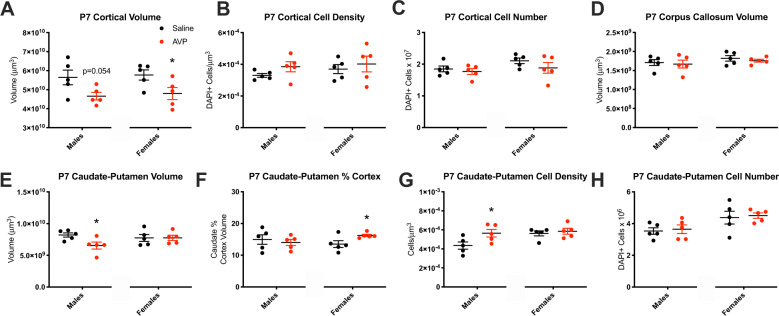
Juvenile offspring neurobiology was altered by maternal AVP. **a** Cortical volume in postnatal day 7 (P7) offspring was trend-smaller in males (*p* = 0.054) and significantly smaller in AVP-exposed females (*p* = 0.047). **b** Cortical cell density and (**c**) cell number were not changed by AVP at P7, (**d**) nor was corpus callosum volume, in either sex. **e** The caudate–putamen was smaller by volume in P7 male AVP-exposed offspring (*p* = 0.032), while (**f**) caudate–putamen volume relative to cortical volume was increased among P7 AVP-exposed females (*p* = 0.043). **g** The density of cells in the caudate was increased in P7 AVP-exposed males (*p* = 0.046), resulting in (**h**) an unchanged number of cells with AVP exposure in either sex. *p < 0.05 per two-sample *t*-test, error bars represent SEM. 2 litters per condition, 2–3 brains per sex per litter.

To trace the neurodevelopmental origins of abnormal behavior and neurobiology in AVP-exposed adult offspring, we next assessed juvenile (P7) offspring brain. Unlike adults, P7 AVP condition female offspring had significantly smaller cortical volumes, while cortical volumes trended smaller in AVP condition males [females: *t*(8) = 2.341, *p* = 0.047, males: *t*(8) = 2.258, *p* = 0.054] (Fig. 3A). Cortical cell density and cell number were not changed at P7, nor was corpus callosum volume, as in adults (Fig. 3B, C, D).

Given adult AVP-exposed female caudate–putamen changes, we also assessed P7 caudate–putamen volume and cell number. Unlike adults, P7 male AVP-exposed offspring exhibited a decrease in caudate–putamen volume [*t*(8) = 2.594, *p* = 0.032] while females were unchanged (Fig. 3E). Notably, as in adults, caudate–putamen volume relative to cortical volume was increased among P7 AVP females [*t*(8) = 2.397, *p* = 0.043] (Fig. 3F), indicating a potential precursor for adult AVP-exposed female enlargements (Fig. 2I). Further assessment revealed increased caudate–putamen cell density in P7 AVP-exposed males [*t*(8) = 2.353, *p* = 0.046] (Fig. 3G), such that the total number of cells in the caudate–putamen was unchanged as in adults (Fig. 3H).

### Embryonic offspring neurobiology

**Fig. 4:**
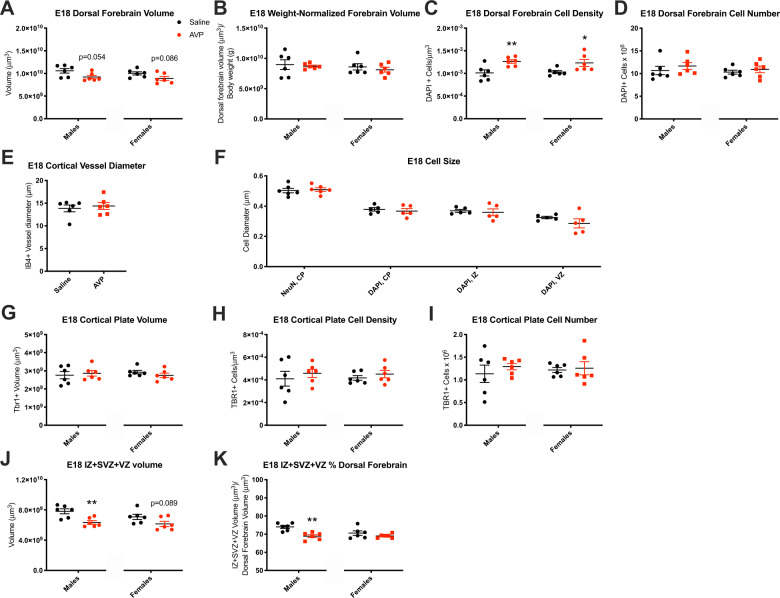
Embryonic offspring neurobiology was altered by maternal AVP. **a** The embryonic day (E) 18 dorsal forebrain was trend-smaller in AVP-condition offspring (males: *p* = 0.054, females: *p* = 0.086), which reflected (**b**) symmetrical growth restriction relative to body weight decrements with AVP exposure. **c** Dorsal forebrain cell density significantly increased with AVP exposure in male and female offspring [males: *p* = 0.009, females: *p* = 0.035] such that (**d**) the total forebrain cell number was unchanged. These volume differences were not accounted for by (**e**) changes in cortical vessel diameter or (**f**) cell size across the cortical plate (CP), intermediate (IZ), or ventricular (VZ) zones. **g** The TBR1-labeled cortical plate volume, (**h**) cell density, (**i**) and cell number were unchanged with AVP. **j** The volume of the intermediate, subventricular, and ventricular zones (IZ + SVZ + VZ) was significantly smaller in E18 AVP-exposed male offspring and trend-smaller in AVP-exposed female offspring (males: *p* = 0.005, females: *p* = 0.089), (**k**) also reflected by a decreased proportion of dorsal forebrain occupied by IZ + SVZ + VZ in AVP-exposed males (*p* = 0.001). *p < 0.05, **p < 0.01 per two-sample *t*-test, error bars represent SEM. 3 litters per condition, 1–3 brains per sex per litter.

E18 offspring neurobiology was evaluated to determine the embryonic origins of postnatal impacts. As at P7, E18 dorsal forebrain (the cortical precursor) was trend-smaller in AVP-condition offspring [males: *t*(10) = 2.184, *p* = 0.054, females: *t*(10) = 1.903, *p* = 0.086] (Fig. 4A). When dorsal forebrain volume was measured relative to body mass, an important analog of brain-symmetrical or asymmetrical human fetal growth deficits^14^, there were no group differences, demonstrating a brain-symmetrical (non-brain-sparing) IUGR phenotype (Fig. 4B). Dorsal forebrain cell density increased with AVP exposure [males: *t*(10) = 3.244, *p* = 0.009, females: *t*(10) = 2.442, *p* = 0.035] (Fig. 4C) such that total cell number in forebrain was unchanged (Fig. 4D) as found postnatally, despite a smaller embryonic dorsal forebrain.

We next assessed whether other contributions to volume might account for the trend E18 dorsal forebrain volume decrements with AVP. One potential contributor is cerebrovasculature. Cortical vessel diameter was unchanged with AVP exposure (Fig. 4E). Furthermore, given no change in dorsal forebrain cell numbers with AVP exposure at E18 (Fig. 4D), we assessed cell size across multiple dorsal forebrain subregions. Terminally-differentiated, NeuN+ cell diameter in the cortical plate was not changed, nor was the diameter of DAPI + cells in other subregions (Fig. 4F). Collectively, these results demonstrate that dorsal forebrain decrements at E18 with AVP were likely due to extracellular changes, rather than changes to cerebrovasculature or to cells themselves (number or size).

Unlike the dorsal forebrain overall, the cortical plate volume, defined here with TBR1 immunohistochemical labeling, was unchanged with AVP exposure (Fig. 4G). Cortical plate TBR1 + cell density and number were likewise unchanged with AVP (Fig. 4H, I).

Given that decreased dorsal forebrain volume was not accounted for by decreased cell number, cell size (Fig. 4D, F), or cortical plate volume (Fig. 4G), we assessed intermediate, subventricular, and ventricular zone volume. The volume of these transitory embryonic brain regions was significantly smaller in E18 AVP-exposed male offspring and trend-smaller in AVP-exposed female offspring [males: *t*(10) = 3.606, *p* = 0.005, females: *t*(10) = 1.880, *p* = 0.089] (Fig. 4J). This was also reflected in the proportion of dorsal forebrain occupied by the IZ + SVZ + VZ, which was significantly decreased in AVP-exposed males [*t*(10) = 4.349, *p* = 0.001] (Fig. 4K). In summary, E18 volume assessments revealed that dorsal forebrain was smaller with AVP exposure despite unchanged dorsal forebrain cell number. This impact occurred not in cortical plate but in the transitory intermediate, subventricular, and ventricular zones. At E14, dorsal forebrain volume differences were not present (saline: 1.64 × 10^9^ ± 6.90 × 10^7^ μm^3^, AVP: 1.48 × 10^9^ ± 7.25 × 10^7^ μm^3^; *n* = 6, 5 from 3, 4 respective litters).

### Embryonic offspring transcriptomics

To understand this impact of maternal AVP administration on E18 offspring dorsal forebrain, we also assessed transcriptomic changes. mRNA-seq revealed 49 differentially expressed (DE) genes with AVP exposure (11 down, 38 up) in male dorsal forebrain (Supplementary Table 2) and 31 (1 down, 30 up) in female (*n* = 4 per condition per sex except *n* = 3 for female AVP, excluding one outlier for high variance; Supplementary Table 3 and Supplementary Fig. 4). Among the top five DE genes by fold change were genes involved in development (males: *Capn11, Slc26a4*; females: *Cped1, Gprc5c, Postn, Mrc1*), neurodifferentiation and neurogenesis (males: *Gdf1, Tfap2d*; females: *Mrc1*), cortical patterning and development (males: *Fgf15*), and immune function (females: *Mrc1, Postn, Cped1, Lox*).

**Fig. 5:**
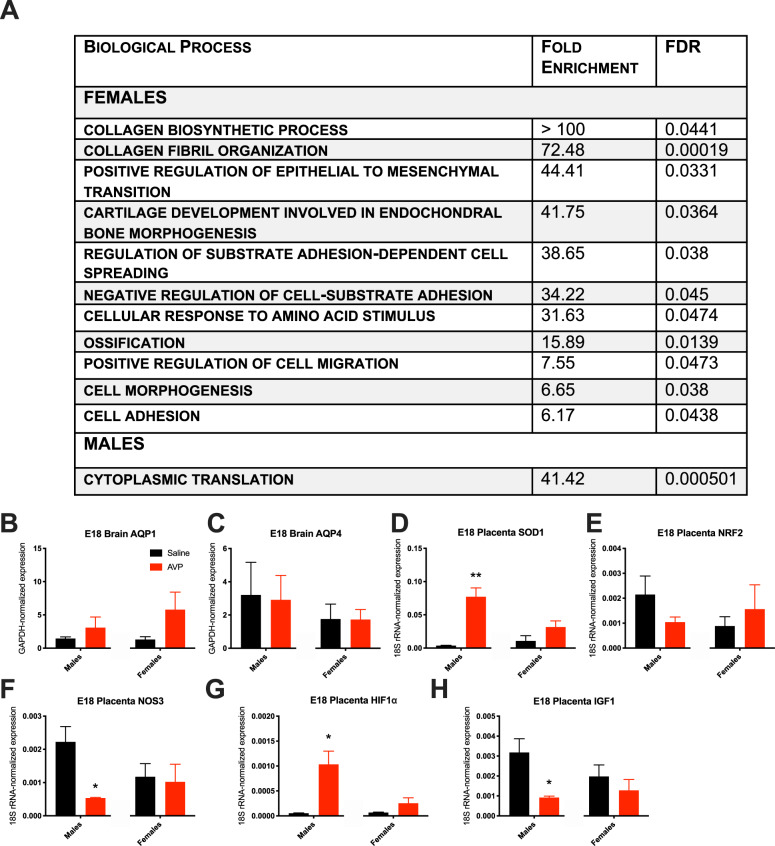
Transcription indicates E18 cortical sex-specific changes and E18 placental male-specific redox and functional changes with maternal AVP. **a** Functional annotation of DE genes with AVP exposure revealed 11 DE biological processes in females and 1 in males with AVP exposure via PANTHERDB. *n* = 4 per group per sex except *n* = 3 for female AVP, excluding one outlier for high variance, each from an independent litter. **b** Embryonic day (E) 18 dorsal forebrain expression of Aquaporin 1 (*AQP1*) and **c** Aquaporin 4 (*AQP4*) were not changed by maternal AVP. **d** Transcript levels of superoxide dismutase (*SOD1*) were significantly increased with AVP exposure in E18 placentas of AVP-exposed males (*p* = 0.005). **e** Levels of erythroid 2–related factor 2 (*NRF2*) transcripts were unchanged. **f** An AVP male-specific decrease occurred in placental nitric oxide synthase 3 (*NOS3*) transcripts (*p* = 0.021). **g** Placental hypoxia-inducible factor 1-alpha (HIF1α) expression increased with AVP exposure in male offspring (*p* = 0.021). **h** Insulin-like growth factor 1 (*IGF1*) expression decreased specifically in males (*p* = 0.031). *p < 0.05, **p < 0.01 per two-sample *t*-test, error bars represent SEM.

Functional annotation (via PANTHERDB) of DE genes with AVP exposure revealed distinct biological processes impacted in males and females (Fig. 5A). In females, extracellular protein-related pathways (e.g., collagen biosynthesis) were altered. In males, the broader “cytoplasmic translation” process was the only one significantly overrepresented by DE genes with AVP exposure.

Finally, to interrogate potential dysregulation of osmotic control by direct AVP action^32^ in the developing brain, E18 dorsal forebrain was assessed for altered aquaporins. Neither aquaporin 1 (*Aqp1*) nor 4 (*Aqp4*) expression was significantly changed by AVP (Fig. 5B, C).

### Placental gene expression

We next assessed E18 placental changes with maternal AVP. Expression of superoxide dismutase (*Sod1*), an endogenous antioxidant enzyme, was significantly increased with AVP exposure in males [*t*(4) = 5.609, *p* = 0.005], while another redox responsive molecule, nuclear factor erythroid 2–related factor 2 (*Nrf2*), was unchanged (Fig. 5D, E). A male-specific decrease occurred in nitric oxide synthase 3 (*Nos3*), which produces the redox-active molecule nitric oxide and is thus involved in angiogenic and vascular regulation [*t*(4) = 3.669, *p* = 0.021] (Fig. 5F). Placental hypoxia-inducible factor 1-alpha (*Hif1*α) expression increased with AVP in male offspring placenta [*t*(4) = 3.689, *p* = 0.021] (Fig. 5G). Insulin-like growth factor 1 (*Igf1*) expression was also decreased specifically in males [*t*(4) = 3.270, *p* = 0.031] (Fig. 5H), indicating broad placental anabolic and growth dysfunction.

To complement our late-gestation assessments here, we performed a secondary analysis of published placental transcriptomics in the same AVP model assessed in mid-gestation (E12.5). Given the link between preeclampsia and ASD and out own autism-relevant neurobehavioral outcomes, we assessed for over-representation of ASD high confidence genes in the DE genes of E12.5 AVP-exposed placenta. Of 87 genes altered by AVP exposure in E12.5 placenta (25 down, 62 up)^15^, six were represented in the SFARI Human Gene Module as high or strong confidence genes (high confidence: *Tcf4, Ebf3, Mboat7*; strong confidence: *Sema5a, Usp15, Zmynd11*) and two as syndromic (*Rorα, Tti2*). This yielded an over-representation of SFARI genes (*p* = 0.023) among those altered by AVP exposure in E12.5 placenta.

## Discussion

Given the large and growing body of research evidencing a risk of preeclampsia to neurodevelopment, it is critical that mechanisms and physiology underlying this risk are studied in animal models^3^. In the present study, we determined that a chronic AVP infusion mouse model, validated for its relevance to preeclampsia^14,15^, resulted in altered offspring neurobiology and behavior.

From a mechanistic perspective, it is revealing that gestational AVP resulted in offspring behavioral changes here. In AVP-exposed male offspring, we found increased sociability and increased anxiety-like behavior on the elevated plus maze, while in females procedural learning was disrupted. Prior studies of the effect of gestational vasopressin administration on offspring behavior in rats have also revealed changes in memory-related processes, including reduced memory retrieval in male offspring^33^, enhanced retention in female offspring^34^, and better cue-discrimination in both sexes^35^. Differences in memory outcomes (deficits here vs enhancements reported previously) may be related to the model species used (rats vs mice) and AVP administration protocol differences (throughout gestation here, only late-gestation previously), as well as differing behavioral endpoints (rotarod and y-maze here vs foot-shock motivated brightness discrimination and passive avoidance tasks previously). The early and continuous gestational exposure to AVP employed in the present study which recapitulates early AVP changes in clinical preeclampsia may also illicit changes to downstream processes, including the oxidative stress and pro-inflammatory processes^15,16^, that are not otherwise initiated with a later, more abbreviated exposure.

Despite methodological differences, both our work and previous studies demonstrate that chronically increased maternal vasopressin is sufficient to cause sustained and significant changes to offspring brain function. Changes to anxiety and memory-related behaviors have also been noted in other animal models of preeclampsia, including the L-NAME treatment^36,37^ and PlGF deficiency models^38,39^. Here, we have expanded upon prior findings by also addressing potential neuromolecular and neurocellular changes in this model—sex-specific abnormalities in cortical and subcortical growth, and synaptic protein distribution, as well as embryonic brain transcriptomics.

The PlGF deficiency and L-NAME treatment models both specifically exhibited abnormal spatial memory^37,38^, coupled in the latter case with altered hippocampal neurogenesis^36^. Collectively, this work suggests common disruptions to memory neurocircuitry, as demonstrated by abnormal behavior and hippocampal synaptic densities in AVP-exposed female offspring here. Prior studies in animal models of gestational pro-inflammation, which also occurs in the L-NAME^40^ and AVP models and in clinical preeclampsia^16^, suggest a potential mechanism—maternal immune dysregulation may drive abnormalities in offspring hippocampal synaptic physiology^41,42^ and memory dysfunction^43^. AVP and its other impacts on physiology that overlap with L-NAME and PlGF exposure may therefore be mechanistically central to memory outcomes, though future work should more carefully examine the mechanisms by which impacts on different behavioral domains are changed.

AVP model effects on offspring behavior and the clinical literature overlap in critical aspects. For instance, offspring intelligence^8^, memory^44^, reasoning^45^, and sociability^46,47^ are negatively impacted by prenatal preeclampsia exposure. The increased sociability in males in this AVP model may not appear directly analogous to the social deficit of ASD disorder for which preeclampsia exposure is a risk. But dysregulated social behavior in either direction may reflect similar disruptions of the circuitry underlying these functions. Given that we did not test female offspring sociability here, future studies should address this to determine sex-specificity of this AVP model on social behavior. Furthermore, the sex-specific results of preeclampsia exposure on children’s neuropsychiatric outcomes remain largely unclear, as few studies have explored this. Some work indicates increased resilience in male offspring, for instance to psychiatric disease^48^ or mood disorders^49^ after preeclampsia exposure. However, other work shows cognitive impacts in men decades after preeclampsia exposure^10^. The precise role of sex as a biological variable in neurodevelopmental programming by preeclampsia exposure will require more in-depth clinical and preclinical studies.

In addition to the sex-specific changes to memory systems resulting from prenatal maternal AVP administration, we also found sex- and development-specific impacts of AVP exposure on the caudate–putamen. At P7, AVP-exposed male offspring had decreased caudate–putamen volume, in line with reductions in other regions, while females had enlarged caudate–putamen volume relative to cortical volume. By adulthood, these male deficits resolved, while female caudate–putamen enlargement persisted. This change in the caudate–putamen, which is a neural substrate of procedural learning, aligned here with the procedural memory deficits in AVP-exposed adult female offspring—increased caudate–putamen cell numbers were inversely correlated with post-training rotarod performance in females. Other models of prenatal challenge, such as the prenatal zinc deficiency model, also result in offspring procedural learning deficits^50^ and striatal enlargement^51^. Similarly, genetic mouse models of ASD, as well as human ASD, are associated with increased striatal volume relative to total brain volume^52^. Abnormal offspring caudate growth may also be a feature of clinical preeclampsia, with neuroimaging revealing increased caudate volume and fractional anisotropy in preeclampsia-exposed children^11^, but the sex-specificity of these outcomes remains unclear.

Our studies in the AVP model also suggest developmental impacts to the cortex. RNA sequencing of E18 primordial cortex (dorsal forebrain) revealed dysregulation of key neurodevelopment genes (*GDF1, TFAP2D*, *MRC1*, and *FGF15*) with AVP exposure. Dorsal forebrain volume at E18 was trend-decreased (cortical volume at P7 and in adulthood was unchanged), though cell number was preserved due to an increased cellular packing density. Further investigation revealed that the cortical plate was unaffected but that the intermediate, subventricular, and ventricular zones were smaller, particularly in males. Of interest, male transcriptomics also showed dysregulated translation, a mechanism implicated by other autism models^53^ and a potential source of reduced dorsal forebrain volume. This volume decrease was not explained by diminished cell numbers, cell size, or cerebrovascular deficits, indicating that extracellular structure may play a role. Though AVP serves osmotic control functions^32^, our assessment of osmotic dysregulation in the offspring brain (*Aqp1* and *Aqp4*) did not demonstrate a role for this at the transcriptional level as a cause of volume dysregulation. The integrity of other potential regulators of brain water content, including the lymphatic drainage system and the blood–brain barrier, should be also examined in futures studies of this model^54,55^. Despite no changes to *Aqp1* or *4* gene expression here, RNA sequencing revealed dysregulation of extracellular collagen-related gene expression in females. Future studies should examine the sex-specificity of these transcriptional changes, and the nature of extracellular brain deficits in the AVP model using more targeted approaches (e.g., proteomics).

It is important that models closely approximate their relevant clinical condition. AVP-infused dams had significantly higher urine protein at E18, as has been noted previously in this AVP model [at E12.5:^15^, at E17:^14^], and placental oxidative stress, as in clinical preeclampsia^56^ and other preeclampsia models^57^. Furthermore, late-gestation IUGR demonstrated here by decreased pup weight at E18 aligns well with the preeclampsia literature^58^ and has been demonstrated previously in this model^15^ and in another AVP model involving late-gestation vasopressin administration in rats^59^. While preeclampsia is diagnosed only after the 20^th^ week of gestation and AVP administration begins slightly prior to conception in the AVP model, this too supports its translational value. Early administration of AVP replicates the clinical finding that AVP is elevated as early the first trimester in pregnancies that eventually develop preeclampsia^14^. Other elements of preeclampsia pathoetiology are similarly at play pre- or peri-conception, including immune dysregulation^60^ and abnormal nonpregnant cardiovascular physiology^61^. Furthermore, AVP at the dose used here causes hypertension only in pregnant animals^14^ and must be administered throughout all of pregnancy (as opposed to only through early or middle pregnancy) to elicit significant hypertension, proteinuria, and fetal growth restriction phenotypes^15^. Administration of AVP just prior to conception and chronically throughout gestation in this model thus disrupts maternal physiology in its earliest stages, as many have argued occurs in clinical preeclampsia, and elicits critical, translationally-relevant preeclampsia phenotypes.

While some modeled preeclampsia leads to brain-sparing, asymmetrical IUGR [e.g., with prenatal inflammation^62^], others models [e.g., prenatal AVP, hypertension, and reduced placental blood flow in rats^36^] lead to symmetrical, brain-impacting growth restriction. Clinically, severe preeclampsia reduces child head circumference relative to body length and is thus not brain-sparing, while less severe gestational hypertension has the opposite effect^58^. The AVP model used here may, therefore, recapitulate the non-brain-sparing symmetrical IUGR of more severe disease.

It is unclear whether sexual dimorphism of the brain itself during its early development is critical for sex-specific impacts in this model at E18. However, the placenta is highly sexually dimorphic^63^ and may underlie such impacts. Male offspring vulnerability was shown at the level of the placenta at E18. Male-specific placental redox dysfunction (*Sod1*), angiogenic and growth factor dysfunction (*Nos3* and *Igf1*), and hypoxia (*Hif1*α) were suggested. Interestingly, transcriptomics in the AVP model placenta previously revealed oxidative stress, but not hypoxia, though placentas were from mid-gestation (E12.5) and were pooled across sexes^15^. Hif1α increased expression here may demonstrate impacts of AVP-induced physiological stress or even hypoxia in the context of late-gestation placental demands which are absent earlier. Further study is required to determine AVP’s temporal and sex-specific impacts to hypoxia processes and whether late-stage hypoxic environment or another *Hif1*α-effector is responsible. Redox dysregulation may be caused by ischemia and decreased antioxidant production in clinical preeclampsia^64^ and may prime the fetal brain for later dysfunction, as in other prenatal insult models^65^. Further tracing pathways changed by preeclampsia in a coordinated way from placenta to the developing brain may provide insights into shifts in the placenta–brain axis, a critical mediator of neurodevelopment^66^.

ASD-related gene regulation in the placenta may also alter offspring neurodevelopment. Our secondary analyses of a previously published RNA sequencing dataset from the E12.5 placenta of this same model revealed over-representation of ASD genes^15^. It should be noted, however, that many of the genes which overlap between AVP placenta transcriptomics and the SFARI dataset have broad functionality (e.g., *Tcf4*, *Mboat7*, *Sema5a*, *Usp15*, and *Rorα* in the immune system) and may thus be disrupted in multiple disorders linked to gestational exposures. Because of the early developmental origins of ASD and its links with pregnancy conditions, exploration of ASD gene functions in placenta may be an important future area of study for revealing pathogenic processes.

In summary, the present study reveals that chronic gestational AVP, modeling preeclampsia, affects the developing brain. Gestational AVP resulted in sex-specific changes to offspring, including to prenatal neurobiology and placenta and postnatal synapses, caudate–putamen volume, and behavior. Our results indicate potential targets for intervention, including extracellular processes in the embryonic brain and oxidative stress and hypoxia in the placenta. As with the clinical preeclampsia literature, this work also suggests that the exposure to preeclampsia is itself a contributing factor for children’s neurodevelopmental and psychiatric problems and that these associations are not the result of other confounds. More closely monitoring neurodevelopmental outcomes in preeclampsia-affected children may thus be a first step in targeting therapy and improving outcomes.

## Supplementary information

Supplementary Fig. 1: Offspring body weight was impacted by maternal AVP.

Supplementary Fig. 2: Open field behavior unchanged by maternal AVP.

Supplementary Fig. 3: Adult regional volumes and cell numbers unchanged by maternal AVP.

Supplementary Fig. 4: Dorsal forebrain mRNA sequencing results for AVP- and Saline-condition E18 offspring.

Supplementary Table 1

Supplementary Table 2

Supplementary Table 3

Supplementary figure, table legends

## Data Availability

All code is available from the authors upon reasonable request.
