# Supplementary figures and images for: Altered offspring neurodevelopment in an arginine vasopressin preeclampsia model
Source: Transl Psychiatry. 2021 Jan 28;11:79. doi: 10.1038/s41398-021-01205-0 (PMC7844013; doi:10.1038/s41398-021-01205-0)

**A****E14 Body Weight**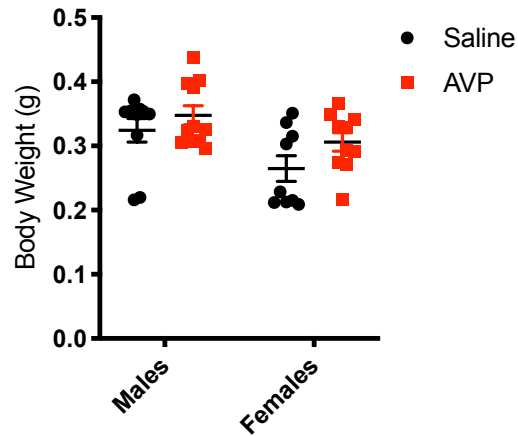**B****E18 Body Weight**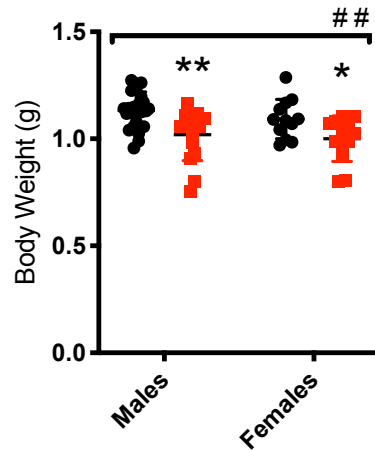**C****P21 Body Weight**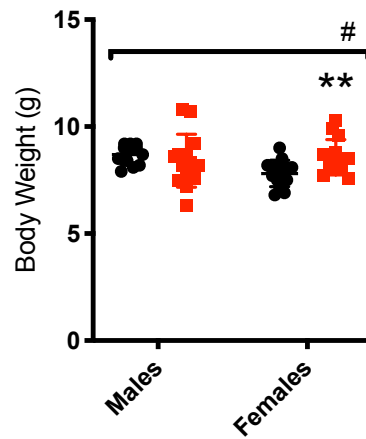**D****Adult Body Weight**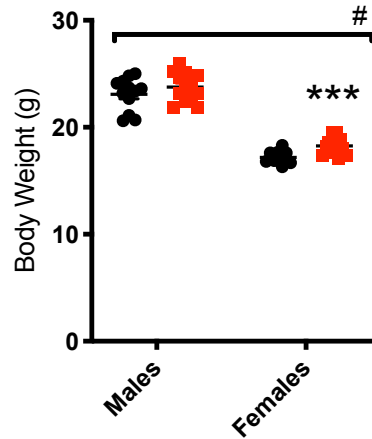

Supplement: Supplementary file 1 — Supplementary Fig. 1: Offspring body weight was impacted by maternal AVP. [file 41398_2021_1205_MOESM1_ESM.pdf]

**A****Open Field Total Distance Traveled**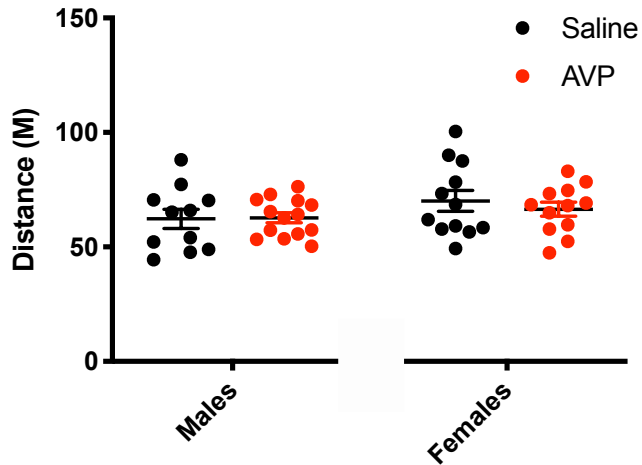**B****Open Field Center Time**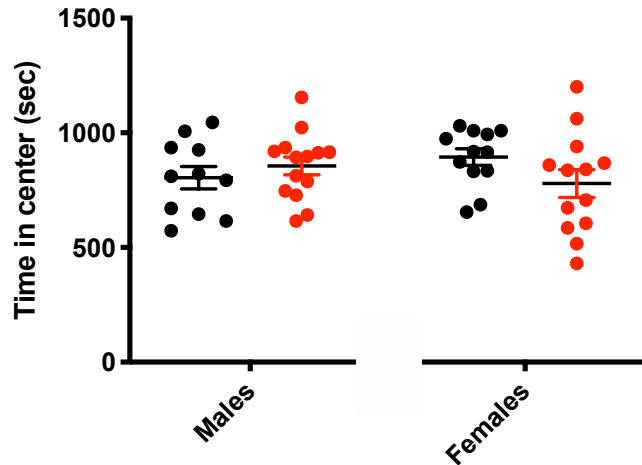

Supplement: Supplementary file 2 — Supplementary Fig. 2: Open field behavior unchanged by maternal AVP. [file 41398_2021_1205_MOESM2_ESM.pdf]
