# Supplementary material for: Altered offspring neurodevelopment in an arginine vasopressin preeclampsia model
Source: Transl Psychiatry. 2021 Jan 28;11:79. doi: 10.1038/s41398-021-01205-0 (PMC7844013; doi:10.1038/s41398-021-01205-0)
Supplement: Supplementary file 3 — Supplementary Fig. 3: Adult regional volumes and cell numbers unchanged by maternal AVP. [file 41398_2021_1205_MOESM3_ESM.pdf]

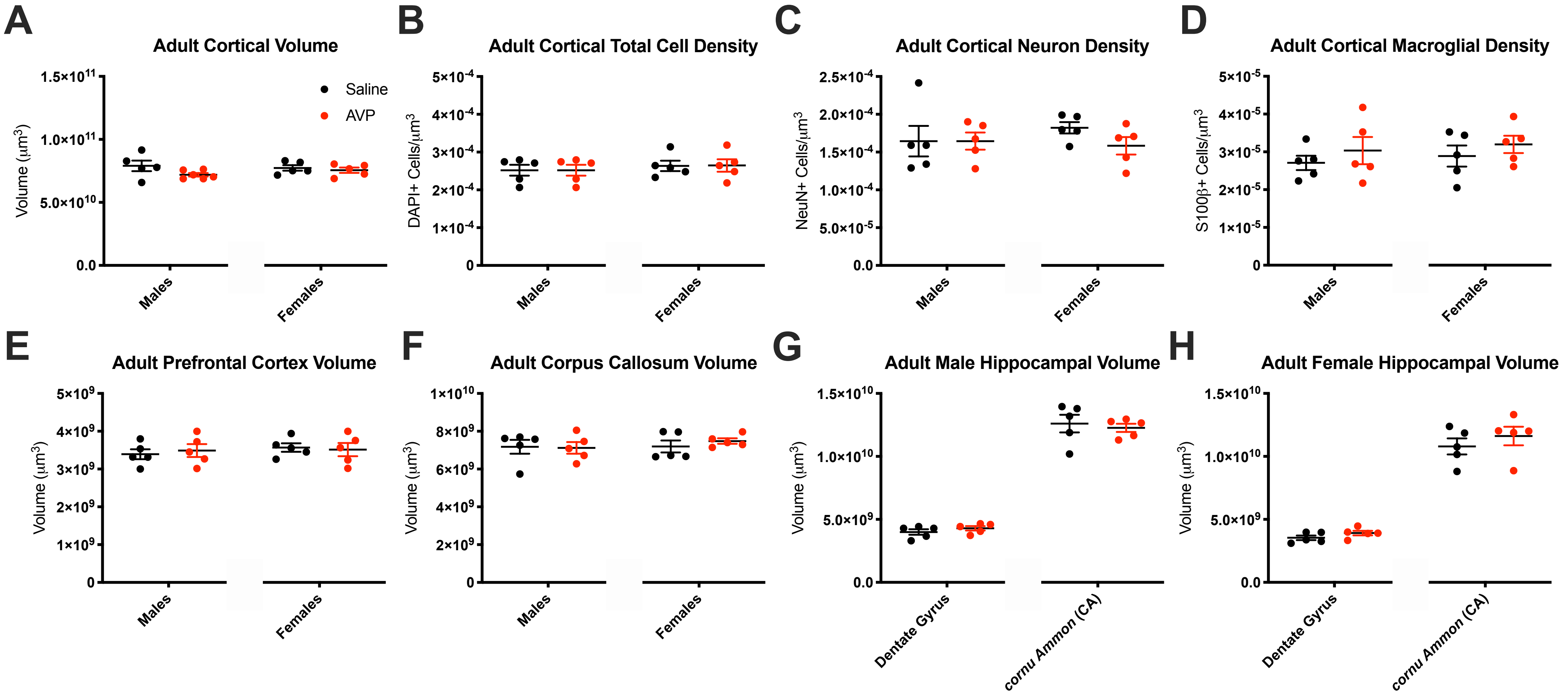

A
Adult Cortical Volume
Saline
AVP
Volume (μm³)
1.5×10¹¹
1.0×10¹¹
5.0×10¹⁰
0.0
Males
Females
B
Adult Cortical Total Cell Density
DAPI+ Cells/μm³
5×10⁻⁴
4×10⁻⁴
3×10⁻⁴
2×10⁻⁴
1×10⁻⁴
0
Males
Females
C
Adult Cortical Neuron Density
NeuN+ Cells/μm³
2.5×10⁻⁴
2.0×10⁻⁴
1.5×10⁻⁴
1.0×10⁻⁴
5.0×10⁻⁵
0.0
Males
Females
D
Adult Cortical Macroglial Density
S100β+ Cells/μm³
5×10⁻⁵
4×10⁻⁵
3×10⁻⁵
2×10⁻⁵
1×10⁻⁵
0
Males
Females
E
Adult Prefrontal Cortex Volume
Volume (μm³)
5×10⁹
4×10⁹
3×10⁹
2×10⁹
1×10⁹
0
Males
Females
F
Adult Corpus Callosum Volume
Volume (μm³)
1×10¹⁰
8×10⁹
6×10⁹
4×10⁹
2×10⁹
0
Males
Females
G
Adult Male Hippocampal Volume
Volume (μm³)
1.5×10¹⁰
1.0×10¹⁰
5.0×10⁹
0.0
Dentate Gyrus
cornu Ammon (CA)
H
Adult Female Hippocampal Volume
Volume (μm³)
1.5×10¹⁰
1.0×10¹⁰
5.0×10⁹
0.0
Dentate Gyrus
cornu Ammon (CA)
