# Supplementary material for: Altered offspring neurodevelopment in an arginine vasopressin preeclampsia model
Source: Transl Psychiatry. 2021 Jan 28;11:79. doi: 10.1038/s41398-021-01205-0 (PMC7844013; doi:10.1038/s41398-021-01205-0)
Supplement: Supplementary file 4 — Supplementary Fig. 4: Dorsal forebrain mRNA sequencing results for AVP- and Saline-condition E18 offspring. [file 41398_2021_1205_MOESM4_ESM.pdf]

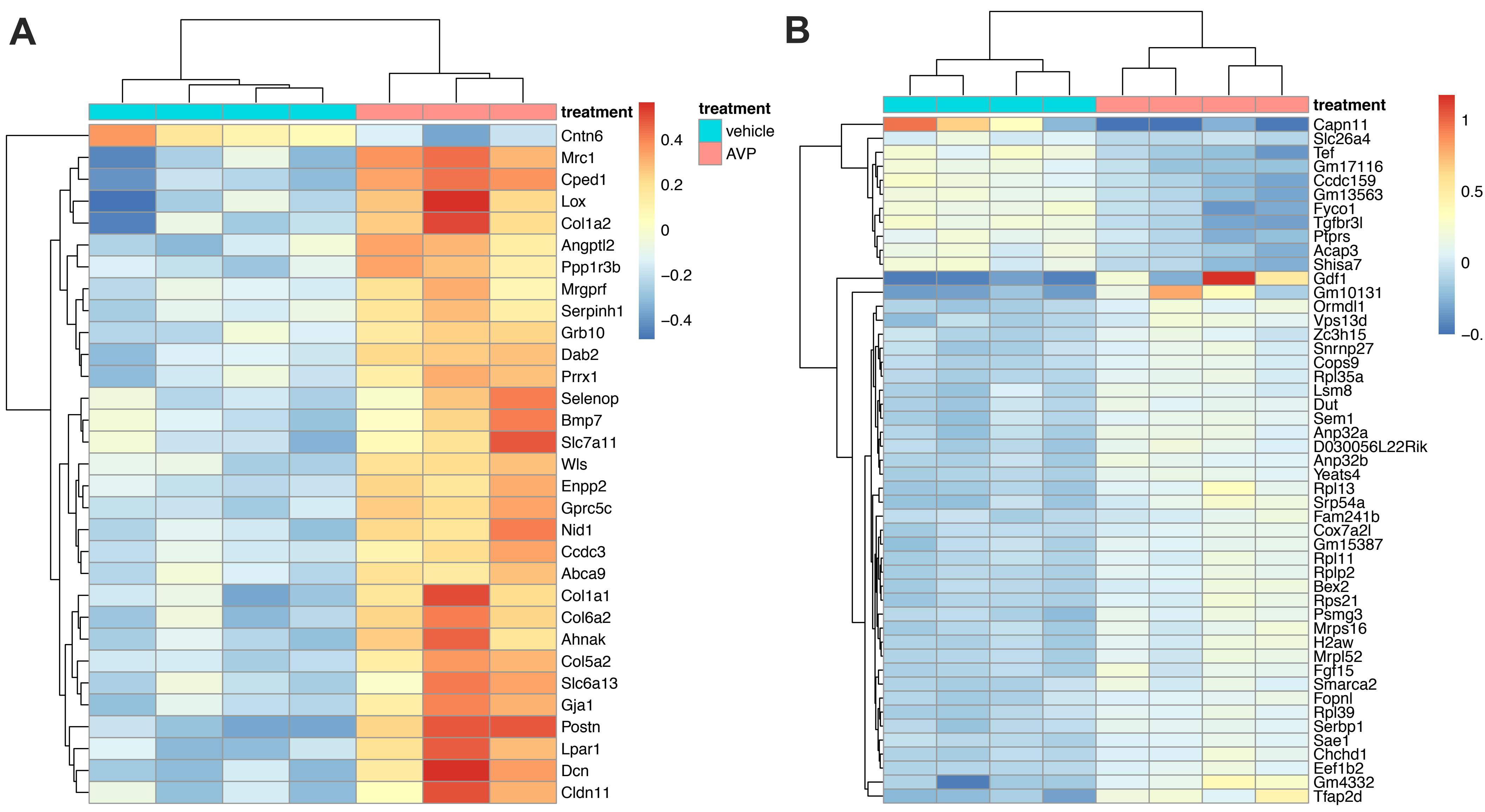

A
B
treatment
vehicle
AVP
0.4
0.2
0
−0.2
−0.4
Cntn6
Mrc1
Cped1
Lox
Col1a2
Angptl2
Ppp1r3b
Mrgprf
Serpinh1
Grb10
Dab2
Prrx1
Selenop
Bmp7
Slc7a11
Wls
Enpp2
Gprc5c
Nid1
Ccdc3
Abca9
Col1a1
Col6a2
Ahnak
Col5a2
Slc6a13
Gja1
Postn
Lpar1
Dcn
Cldn11
1
0.5
0
Capn11
Slc26a4
Tef
Gm17116
Ccdc159
Gm13563
Fyco1
Tgfbr3l
Ptprs
Acap3
Shisa7
Gdf1
Gm10131
Ormdl1
Vps13d
Zc3h15
Snrnp27
Cops9
Rpl35a
Lsm8
Dut
Sem1
Anp32a
D030056L22Rik
Anp32b
Yeats4
Rpl13
Srp54a
Fam241b
Cox7a2l
Gm15387
Rpl11
Rplp2
Bex2
Rps21
Psmg3
Mrps16
H2aw
Mrpl52
Fgf15
Smarca2
Fopnl
Rpl39
Serbp1
Sae1
Chchd1
Eef1b2
Gm4332
Tfap2d
