# Supplementary material for: Altered offspring neurodevelopment in an arginine vasopressin preeclampsia model
Source: Transl Psychiatry. 2021 Jan 28;11:79. doi: 10.1038/s41398-021-01205-0 (PMC7844013; doi:10.1038/s41398-021-01205-0)
Supplement: Supplementary file 8 — Supplementary figure, table legends [file 41398_2021_1205_MOESM8_ESM.docx]

**Supplementary Figure legends:**

**Supplementary Fig. 1: Offspring body weight was impacted by maternal AVP.**

A) Embryonic day (E) 14 body weight was unchanged by maternal AVP infusion. B) At E18, AVP-exposed offspring were smaller (main effect of AVP by two-way ANOVA p<0.001; posthoc t-test males: p=0.0018; females: p=0.033, corrected alpha=0.025). C) At P21, female AVP-exposed offspring were larger than saline comparators (interaction by two-way ANOVA p=0.029; posthoc t-test females: p=0.011; males: p=0.46, corrected alpha=0.025). D) As adults, AVP-exposed females remained larger and males were unchanged (main effect of AVP by two-way ANOVA p=0.0097; posthoc t-test females p=0.0007; males: p=0.26, corrected alpha=0.02). ^#^p<0.05, ^##^p<0.001 by two-way ANOVA. *p<0.05, **p<0.01, ***p<0.001 per two-sample t-test, error bars represent SEM.

**Supplementary Fig. 2: Open field behavior unchanged by maternal AVP.**

A) Total distance traveled and (B) time spent in the center of the open field were unchanged in male and female adult offspring.

**Supplementary Fig. 3: Adult regional volumes and cell numbers unchanged by maternal AVP.**

A-D) Adult cortical volume and the total cell, neuronal, and macroglial cell densities in the cortex were not changed by AVP exposure in male or female offspring. E-H) Prefrontal cortex, corpus callosum, and hippocampal [dentate gyrus (DG) and CA1 and 3] volumes were also unchanged by AVP exposure. n=2-3 litters per sex per condition.

**Supplementary Fig. 4: Dorsal forebrain mRNA sequencing results for AVP- and Saline-condition E18 offspring.**

A) Heat plot or mRNA sequencing results (red=upregulated, blue=downregulated) representing the 31 (1 down, 30 up) differentially-expressed (DE) genes in E18 females and B) 49 DE genes (11 down, 38 up) in E18 males in the dorsal forebrain after maternal AVP administration. n=4 per group per sex except n=3 for female AVP, excluding one outlier for high variance.

**Supplementary Table legends:**

**Supplementary Table 1:** Primers used for qPCR.

**Supplementary Table 2:** RNA sequencing results for comparison of male AVP and Saline condition embryonic dorsal forebrain.

**Supplementary Table 3:** RNA sequencing results for comparison of female AVP and Saline condition embryonic dorsal forebrain.
